# Exploring the effect of professional experience on knowledge towards geriatric care among nurses working in adult care units

**DOI:** 10.1186/s12877-021-02156-3

**Published:** 2021-04-06

**Authors:** Erkihun Tadesse Amsalu, Tesfaye Alemayehu Messele, Metadel Adane

**Affiliations:** 1 Department of Epidemiology and Biostatistics, School of Public Health, College of Medicine and Health Sciences, Wollo University, Dessie, Ethiopia; 2 Amara regional health Bureau, Bahr-Dar, Ethiopia; 3 Department of Environmental Health, College of Medicine and Health Sciences, Wollo University, Dessie, Ethiopia

**Keywords:** Knowledge, Nurses, Geriatric care, Adult care unit, Ethiopia

## Abstract

**Background:**

The elder population suffered from social, economic, and health (which includes physical) related problems. Thus, these problems are complex and interrelated, thereby requiring specific knowledge and expertise to meet them. However, there were limited researches previously done to explore nurse’s knowledge towards geriatric care. Therefore, this study aimed to assess knowledge towards geriatric care and to examine its predictors in North east Ethiopia. The findings will be helpful to develop strategies that would promote nurses’ knowledge, which in turn improves the quality of patient care and consequently, the health of older people.

**Methods:**

A facility-based cross-sectional study was conducted from March 8 to 28, 2020, among 335 nurses. Simple random sampling technique was employed to select study participants. A structured self-administered questionnaire was used to collect data on knowledge of nurses towards older adult care. A multivariable logistic regression model was applied to identify significant predictors and *P*-value< 0.05 was used to declare the significance of association.

**Results:**

In the study, a total of 335 nurses participated. About 192(57.3%) of them had poor knowledge towards older adult care. Thus, level of education (AOR = 1.9, 95%CI: 1.1–3.2), year of experience 1–5 year (AOR = 2.7, 95%CI: 1.5–4.9), 5–10 years (AOR = 2.5, 95%CI: 1.4–4.4), and previously living with older adult at home (AOR = 1.6, 95%CI: 1.05–2.7) were significant predictors of knowledge on older adult care.

**Conclusions:**

Our study showed; most of nurses had poor knowledge of the care of elder patients. Level of education, level of experience, and lived with the older adult were factors that contributed for poor knowledge. Thus, professional organizations should focus on changing nursing curricula and providing professional development workshops to improve the knowledge of nurses on geriatric care.

## Background

The united nation (UN) defines, elder people are those people whose age is 60 years and above [1]. Elder age is the sum of changes over time through a person’s life and universal character that all human beings manifest [2].

Globally elder population was 962 million in 2017. This number will be expected to double again by 2050, which reaches nearly 2.1 billion [3].

African older population have been also increasing rapidly for the next years, in 2005 Africa had 47 million, and between 2000 to 2015 increases by 50%, and after 2050 it increases by five-times [4].

The definition of elder care in Ethiopia also adopted from the UN definition that is 60 years and above as it coincides with the country’s official retirement age [5]. The elder population increase from 3.1 to 5% in 2019 and this proportion is expected to nearly double to 9% by 2050 [5, 6].

As a result elders were more risky to non-communicable disease (NCD) mainly hypertension (HTN), Diabetes Mellitus (DM), eye problems, cardiac problem and arthritis [7]. The increasing or high number of social, economic, and health problems met by older persons requires attention of all concerned bodies to meet their needs and interests [8]. Furthermore, it increases pressures in the health facilities to meet the care of elder people [3].

Geriatric Knowledge is a theoretical and practical understanding of elder care through experience or education of health care providers [9]. Nurses are fully responsible for providing direct care to older persons [10, 11]. They must have geriatric knowledge to understand older people’s complex problems, identify their needs, plan and provide high quality care [11].

Evidence gathered in different countries indicates that nurses had limited geriatric knowledge. A cross- sectional study conducted among nursing students in Zanzibar reported that majority of the participants (82.4%) had poor knowledge on elderly care [12]. Results from the studies reviewed by Liu et al. (2013) indicate that nurses and nursing students have low to average knowledge levels with regards to physical, psychological, and social aspects of aging and key clinical areas of geriatric nursing care [13].

The descriptive cross-sectional study conducted in Nigeria and Egypt also showed that 40 and 72.8% of nurses had poor knowledge on older adult care [14, 15].

A cross-sectional study conducted in Ethiopia on knowledge towards care of elder patients and associated factors among nurses also revealed that the majority of nurses (71.3%) had poor knowledge on elderly care [16].

Previous studies mentioned several predictors that bring poor geriatric knowledge by nurses. These are mainly the educational status of nurses, type of learning program (regular or extension), study elder care content, the experience of living with older adult at home previously, and giving home care for elders [10, 13, 15, 16]. In addition, studies demonstrated a strong and positive significant association between the working unit and the knowledge of nurses towards elder care [17]. Similarly, studies showed a strong positive significant association between knowledge on elderly care and age of nurses [10, 16, 17]. Furthermore, studies reported a strong significant association between marital status and knowledge of nurses towards older adult care [16, 18].

Inadequate knowledge significantly affects the nursing care provision to elder patient. Therefore, there is a growing demand for nurses to improve their knowledge and commitment to work with older people in diverse settings in the context of a rapidly ageing population [19].

Previous studies have shown that adequately prepared nurses with better knowledge and skills towards older adults can improve patient outcomes such as reduced hospital length of stay, reduced readmission rates and increased patient and family satisfaction [20, 21]. In Ethiopia, the care of elder people are not addressed adequately due to the fact that lack of trained personnel, and well organized geriatric care unit and training institutions in the country [16]. In addition, there is no study conducted in the region to assess the knowledge of nurses on older adult care and to examine its predictors.

In response to the limited evidence in the country and to fill this gap in thescientific literature, this study aimed 1) to assess knowledge towards geriatric care and 2) to examine its predictors among nurses working in adult care units of public hospitals in North east Ethiopia. Thus, the findings of this study will be helpful to design strategies for promoting the health of elder people and improving the quality of care of the elder patients.

## Methods

### Study setting and design

A facility- based cross-sectional study was conducted on nurses working in adult care units in North West Ethiopia from March 8 to 28, 2020, to assess knowledge towards geriatric care and its predictors. This design follows the intended use of the KOP-Q as a single self- assessment measurement at one point in time to determine the knowledge levels of nursing working in adult care admission unit.

The in-person survey using well design questionnaire from the point of view of respondents were used as to collect data to improve the response rate.

The study was conducted in Bahr-Dar city, which is the administrative city of Amara national regional state which covers the largest geographical area in Ethiopia. The area is located 572 KM north of Addis Ababa, the capital city of Ethiopia. There are three government hospitals i.e. Felege-Hiwot referral hospital, Tibebe-Ghion Comprehensive specialized teaching Hospital, and Addis-Alem Hospital. And the participants of the study was obtained from three hospitals and working in the adult admission care unit.

The hospitals provide service for about 25 million people. In Ethiopia, the number of elder population has been increasing over time and about 75% of the elder people were suffering from at least one chronic disease and of these 77.5% of them were undergoing medical treatment.

There are about with 500 professional nurses holding diploma, Bachelor degree and master degree providing different service in the adult admission care unit.

### Population

In this study, all nurses who were working in adult care units of three governmental hospitals in Bahr- Dar city was the target population, and the study population includes nurses who were working in the adult care units of three governmental hospitals in Bahr- Dar city during the data collection period. Nurses who were working in the adult admission care units (emergency, ICU and inpatient department) who provided care for elder patients for a minimum of one year were included in the study.

### Sample size determination and sampling technique

The sample size for prevalence was determined based on a single population proportion formula by taking the prevalence of poor knowledge of nurses towards elder care (71.3%) from a previous study [16]. Thus, by considering 5% margin of error (d), 95% confidence level (Z) the sample size was calculated as follows,

$$ N=\frac{\left(\frac{Z\alpha}{2}\right)2.\mathrm{P}.\mathrm{Q}}{(d)^2}=\frac{(1.96)2\left(0.713\times 0.287\right)}{(0.05)2}=316 $$.

Where, Z = 1.96, d = 5%, *P* = 71.3% and Q = 28.7%.

By adding 10% nonresponse rate, the sample size was 348.

The sample size for the second objective was determined based on double population proportion formula using EPI -info version 4.6.0.2 software by considering factors from previous works [19] (Table 1).

**Table 1:** sample size determination for factors associated with knowledge towards elder care by using double population proportion formula

No	Factors	Power	Ratio	CI	Outcome in %		AOR	Sample size
					Unexposed	Exposed		
1	Level of education	80%	1:1	95%	23	54.8	4	86
2	Year of Experience	80%	1:1	95%	15.4	48.2	2.4	114
3	Marital status	80%	1:1	95%	15.9	35.8	2.4	260

Thus, the largest sample size (i.e. 348) obtained from a single population proportion formula was considered as the final sample size of the study.

There were 500 nurses working in the adult care unit in the selected hospitals (Felge Hiwot referral hospital, Tibebe Ghion teaching hospital, and Addis Alem primary hospital). Then proportional allocation to sample size was done in each hospital. Then a simple random sampling technique using lottery method was employed to select the nurses from each hospital. Accordingly, 170 nurses from Felege Hiwot Referral hospital, 140 from Tibebe Ghion teaching hospital, and 38 from Addis Alem primary hospital were included in the study.

### Study variables and operational definition

Knowledge was the outcome variable of the study. Independent variables of the study include socio-demographic factors (sex, age, marital status, work experience, level of education, and living with the elderly), professional-related factors (the type of learning program either regular or extension, type of learning institution either private or government, communication with patient/caregiver family, following elder care policy /guidelines, pre-employment training, special training in elder care, hospital educational support, nursing home visit, and get a reward), and institutional- related factors (working unit, adequacy of workspace/room, and geriatric ward).

In this study Geriatric Knowledge was defined as a theoretical and practical understanding of nurses on elder care through experience or education [22, 23].

Respondents with a KOP-Q score of ≥23 were considered as having good knowledge towards elder care and those respondents with a KOP-Q score < 23 were classified as having poor knowledge on older adult care [16, 22]. In this study, geriatrics refers to those people whose age is 60 years and more [11].

### Data collection instrument and quality assurance

Self-administered English version questionnaire was used to assess nurses’ knowledge on older adult care and its predictors.

To measure knowledge, the KOP-Q was used. The KOP-Q, which was developed and validated in the Netherlands, has a clearly described theoretical basis finding its origin in nursing care knowledge regarding older patients and has good content and construct validity results [22–24]. The tool consists of 30 items that are scored on a dichotomous true/false scale with every correct answer receiving one point, and every incorrect answer receiving zero points (total score: minimum = 0, maximum = 30). The knowledge score for each participant was the sum of the correct answers. Respondents with a KOP-Q score of greater than or equal to 23 were considered as having good knowledge towards elder care and those respondents with a KOP-Q score less than 23 were classified as having poor knowledge on older adult care [16, 22].

The KOP-Q demonstrated adequate face validity, good readability, a good Scale-Content Validity Index/average (S-CVI/ave. = 0.91), and excellent reliability for the certainty items (Cronbach Alpha = 0.94) [23].

Three diploma nurse data collectors and three BSc nurse supervisors were recruited for data collection. Data quality was assured before, during, and after the data collection process. A pretest was done in Gimjabet hospital before actual data collection on 5% of the sample to check the reliability of the tool. Two days of training was given for data collectors and supervisors. There was a close day-to-day supervision of data collectors in the data collection process. After data collection, it was checked for completeness and consistency each day before transferring it into computer software.

### Statistical analysis

The data was coded after checking its completeness. Epi- data version 4.6.0.2 software was used for data entry and SPSS version 25 for further analysis. Descriptive statistics, including frequencies and proportions were employed to present relevant variables. Further, chi-square test was conducted to compare the proportions of poor knowledge of nurses with different demographic variables of participant.

Furthermore, all variables with a *p*-value of < 0.05 were considered to be significant and entered in multivariable logistic regression to determine the predictors of knowledge level towards care of older adult among the participants.

### Ethical consideration

Ethical clearance was obtained from the ethical review committee of, College of Medicine and Health science, Wollo University. Official Permission letter was obtained from the Amara National health Bureau. The purposes and the importance of the study were explained to the study participants. Verbal informed consent was secured from each participant after approved by the ethical committee since the study didn’t involve any procedure and had no minimal risk to the participants. Participants were clearly informed about the study and their participation was voluntary. Name of identifiers and any personal identifiers were not included in the study, and confidentiality was kept at all level of the study.

## Results

### Socio-demographic characteristics of participants

From a total of 348 participants to be planned in the study, 335 were completed the questionnaire, yielding the response rate of 96.26%. The socio-demographic characteristics of the nurses employed in the three hospitals were comparable. Among the total respondents, about 161(48.1%) of them were working in Felege Hiwot referral hospital. Regarding the sex of respondents, more than half, 187 (55.8%) of them were female. About, 130(38.8%), of respondents was in the age group of 21–29 years and the mean age of respondents was 33.9 years (SD = 7.9). More than half of respondents, 188(56.11%) were married, and about two-thirds, 221(66%) of them were BSc degree holders. About 137(40.9%) of respondents had more than 10 years of experience in the nursing profession and the mean years of experience was 8.4 years with SD (=2.3).

Furthermore about, 193(57.6%), of respondents were previously lived with older adult at home **(**Table 2**)**.

**Table 2:** Socio-demographic characteristics of participants working in the adult admission unit of public hospitals in Bahr-Dar Town, North West Ethiopia, 2020 (*n* = 335)

No	Variables	Category	Frequency	Percent
1	Hospitalcurrently working	Felege Hiwot	161	48.1
		Tibebe Ghion	134	40
		Addis Alem	40	11.9
2	Sex	Male	148	44.2
		Female	187	55.8
3	Age	21–29	130	38.8
		30–39	121	36.1
		≥40	84	25.1
4	Marital status	Single	140	41.79
		Married	188	56.11
		Divorced	7	2.1
5	Level of education	Diploma	112	33.4
		Degree	221	66
		Masters	2	0.6
6	Ever lived with the elder	Yes	193	57.6
		No	142	42.4
7	Year of experience	1–5	114	34
		5–10	84	25.1
		> 10	137	40.9

### Institutional and professional related factors of respondents

In this study one-fourth of respondents, 84(25.1%) were working on a medical ward and two-thirds of respondents, 180 (53.7%) did not participate in giving home care for the older adult. Regarding elder care content, more than half, 197(52.3%) of the respondents were not taken the geriatric care content and 109(32.5%) of them were happy to study eldercare content. Nearly three-fourths of the respondents, about 246(73.4%), of them attended their education in the regular program, and out of this, 200(59.7%) of them were learned in governmental institutes.

It showed that about 177(52%) of the participants did not follow eldercare policy guidelines when they provide care for older adult. Regarding training, more than half, 220(65.7%) of the participants were not trained on geriatric care and 231(69%) of them did not get geriatric training from their hospital (Table 3).

**Table 3:** Institutional and professional related characteristics of participants working in the adult admission units in Bahr- Dar Town, North West Ethiopia, 2020 (*n* = 335)

No	Variable	Category	Frequency	Percent
1	Follow elder care policy guideline	Yes	158	47.2
		No	177	52.8
2	Worked inadequate space during care provision	Yes	142	42.4
		No	193	57.6
3	Like to communicate with elder patient/family	Yes	226	67.5
		No	109	32.5
4	Give home care for the elderly	Yes	155	46.3
		No	180	53.7
5	Study elder content	Yes	143	42.7
		No	192	57.3
6	Happy studying elder content	Yes	109	74.3
		No	37	25.7
7	Type of learning program	Extension	89	26.6
		Regular	246	73.4
8	Type of learning organization	Government	200	59.7
		Private	135	40.1
9	Like to work after reward	Yes	100	84.0
		No	19	16.0
10.	Type of working unit/ward	Medical	84	25.1
		Surgical	79	23.6
		Emergency	60	17.9
		OR	34	10.1
		Adult ICU	19	5.6
		Gynecology	26	7.8
		Neurology	21	6.3
		Oncology	12	3.6
11	Taken geriatric training at hospital	Yes	115	34.3
		No	220	65.7
12	Getting training	Yes	122	36.4
		No	213	63.6

### Level and predictors of knowledge on older adult care among the study

#### Participants

The current study revealed that most of nurses about, 192(57.30%), of them had poor knowledge on older adult care (95%CI: 51–62%). The results of chi-square test showed that, the level of knowledge on older adult care among participants differed significantly by type of institution, currently being living with older adult at home, age, years of experience, and Level of education.

After applying multivariable logistic regression, predictors of poor level of knowledge were; experience of living with an older adult person at home, level of education, and year of professional experience.

The finding revealed that participants who had diploma in nursing were 1.9 times more likely to have poor knowledge on elder care compared with those participants who had degree and above in nursing profession (AOR = 1.9, 95%CI: 1.1–3.2).

Study participants who had 1–5 years nursing professional experience were 2.7 times more likely to have poor knowledge of elder care compared with those who had more than 10 years of nursing professional experience (AOR = 2.7, 95%CI:1.5–4.9). Similarly, participants who had 6–10 years professional experience in nursing were 2.5 times more likely to have poor knowledge towards elder care compared with those who had greater than 10 years professional experience (AOR = 2.5, 95%CI:1.4–4.4).

Furthermore the result showed that participants who did not live with older adult were 1.6 times more likely to have poor knowledge on elder care compared with those participants who lived with older adult (AOR = 1.6, 95%CI: 1.05–2.7) (Table 4).

**Table 4:** Predictors of knowledge on elderly care among study participants, 2020 (*n* = 335)

Variables	Knowledge on elderly care		COR(95% CI)	AOR(95%CI)
	Poor	Good		
Currently living with elderly at home				
Yes	97(50.5)	97(67.8)	1	1
No	95(49.5)	46(32.2)	2.06(1.3–3.2)	1.6(1.05–2.7) *
Age				
21–29	90(46.9)	40(28.0)	3.3(1.8–5.8)	1.6(0.5–5.5)
30–39	68(35.4)	53(37.0)	1.8(1.07–3.3)	1.1(0.5–2.2)
> 40	34(17.7)	50(35.0)	1	1
Years of Experience				
1–5	78(44.1)	36(25.9)	3.1(1.8–5.27)	2.7(1.5–4.9) **
5–10	58(24.3)	26(15.8)	3.2(1.8–5.7)	2.5(1.4–4.4) **
> 10	56(31.6)	81(58.3)	1	1
Level of education				
Diploma	80(41.7)	32(22.4)	2.5(1.5–4.0)	1.9(1.1–3.2) *
Degree and above	112(58.3)	111(77.6)	1	1
Typeof learning organization				
Government	130(67.7)	70(49.0)	1	1
Private	62(32.3)	73(51.0)	0.45(0.3–0.7)	0.6(0.3–1.03)

*COR* Crude odds ratio, *CI* Confidence interval, *AOR* Adjusted odds ratio, 1: Reference category, _*:_ significant at *p* < 0.05, _**:_ Significant at *p* < 0.001.

## Discussion

The finding of this study showed that most of nurses had poor knowledge on older adult care. This result was higher than studies done in Nigeria 40% [25], Israel 49% [12], Saudi Arabia14% [10], India 24% [26], Portuguese 21% [17], Malaysia 26% [13], and China 41% [27]. The variation might be due to differences in sample size i.e. small number of respondents were involved in Portuguese [17]. In addition this may be related with the variation in socio-demographic characteristics, educational status, level of experience [10, 13, 26, 27]. Furthermore, in Nigeria nurses working in tertiary hospital were involved, in which they have better access for training, and additional facilities that helps to improve the knowledge of nurses on older adult care [25].

The finding of this study was lower than studies conducted in Zanzibar 82.4% [16] and Egypt 72.8% [15]. These might be due to difference in the type of facility [15] and difference in the level of training where most of the participants in the current study were less experienced with little or no geriatric training but in the previous study respondents were Geriatric nurses in better position to provide elder care [16].

The current finding is also consistent with the report from Netherlands [28]. Most nurses working in the hospitals pass the KOP-Q, although a considerable proportion still scores insufficient to extremely poor (ranging from 10.4 to 54.4% in different groups). May be the slight differences are small numbers (5%) of older patients 60+ and therefore less training. These results demonstrate that it is important that more training will be developed so nurses can get their knowledge up to date.

The finding of this study showed that the level of education was positively and significantly associated with knowledge on elderly care. Accordingly, those participants who had diploma in the nursing profession were more likely to have poor knowledge on elderly care compared to participants who had bachelor degrees. This guides nurses to upgrade their education to provide quality older adult care. It is consistent with studies done in Ethiopia [16], Egypt [15], Nigeria [25], Netherlands [28], and China [27]. This significant association might be related with those participants who had bachelor degree in nursing profession were equipped with better professional knowledge since they learn more advanced courses and elder management, which might in turn improve their knowledge on older adult care [10, 16].

The finding of this study also revealed year of professional experience was significantly associated with knowledge towards the care of elder patients. Accordingly, those participants who had 1–5 years of professional experience were more likely to have poor knowledge towards older adult care than those nurses who had greater than 10 years’ of professional experience. Similarly, participants who had 5–10 years’ of professional experience in nursing were more likely to have poor knowledge towards elder care as compared to those participants who had greater than 10 years’ of professional experience in nursing. This finding was consistent with the study done in Addis Ababa, Ethiopia, in which knowledge of nurses towards elder care increases as the professional experiences of nurses increases [16]. It was also consistent with the study conducted in Saudi Arabia [10] and Sweden [29]. This significant association may be explained as nurses who are more experience might have better opportunity to access up-to-date information about the care of an elder patients gradually from their daily observations, practices, and staffs which would help to improve their knowledge on geriatric care [16].

The result of this study also showed that living with older adult was positively associated with knowledge of nurses towards elder care. Those participants who did not live with the older adult were more likely to have poor knowledge towards elder care compared with nurses who lived with older adult. This result was consistent with a study done in Egypt [15] and in China [27]. This significance might be explained as nurses who live with the older adult had more experience in giving care to elders and this could increase their knowledge towards elder care.

Due to the cross-sectional nature of the study, only association was examined and it was difficult to draw conclusions about causality. There was no adequate study done in Ethiopia on this topic, so no adequate evidence was available to discuss the finding with the national context. Since self-administered questionnaires were used to collect data; the study may be subjected to bias from respondents. Furthermore, the finding was not supported with qualitative data to address qualitative perspectives on the determinants of knowledge on geriatric care, it was better if a mixed design was utilized.

### Implications of the study

Older adult age is associated with changes that constitute a gradual decrease in physiological reserves, an increased risk of chronic diseases, and a general decline in the capacity of the individual. Older adult care requires health workers with specific skills associated with an understanding of the biological, psychological, social and cultural theories related to aging. However, many health professionals are often not adequately ready to deal with the healthcare needs of older adults because many existing training curriculum were developed by twentieth century and had focused much on management and treatment of acute infectious diseases which were the world’s most prevailing health problems. Previous studies have shown that adequately prepared nurses with better knowledge, skills and positive attitude towards older adults can improve patient outcomes such as reduced hospital length of stay, reduced readmission rates and increased patient and family satisfaction.

This study was perhaps the first of its kind in this country and the region which focused on determining knowledge of nurses working in adult care unit toward care for older people.

Thus, the current study demonstrates addressing the identified predictors would help to design proper strategies to improve knowledge of nurses on older adult care.

The study could have valuable policy implication to design proper strategies to enhance the knowledge of nurses to provide quality care and improve elder care. The finding has also relevance for professional institutes to focus on changing nursing curricula and providing professional development workshops/trainings to improve the knowledge of nurses on geriatric care. This study could also provide information to next researchers on knowledge of nurses on elder care and its predictors. In general, the poor knowledge of nurses will have a negative impact on the quality of care and on the quality of life of older patients hence it is important that more training will be developed so nurses can get their knowledge up to date.

## Conclusion

This study showed that most of nurses had poor knowledge towards care of elder patients. Accordingly, year of professional experience, educational status of nurses, and living with the elderly were predictors of knowledge towards care of elder patients.

There was no similar study conducted in the area thus it could give information about the level of knowledge of nurses towards geriatric care and helps to identify its predictors to design proper strategies for improvement of geriatric care. The questionnaire used to collect data was up-to-date and standard, which increases the validity and reliability of the study. The findings could be generalized to similar setting and population in other parts of the country since it is community based primary study.

## Data Availability

All the data and materials used in this study are fully available in the manuscript.

## Abbreviations

AOR: Adjusted odds ratio
CI: Confidence interval
COR: Crude odds ratio
DM: Diabete Mellitus
ERC: Ethical Review Committee
GC: Geriatric Care
HTN: Hypertension
ICU: Intensive Care Unit
IPD: Inpatient Department
KOPQ: Knowledge about Older Patient Quiz
OPACS: Older Patient in Acute Care Survey
SD: Standard Deviation
SPSS: Statistical Package for Social Science
SRS: Simple Random Sampling
UN: United Nation

## References

[1] UN (2019). World Population Prospects 2019. Dep Econ Soc Aff.

[2] Efiong M (2015). Knwoldge, attitiude and practice of care of the elderly pationts among health workers in Nigeria.

[3] UN (2017). World Population Ageing.

[4] Ramathirtham G, Balamurugan J (2012). Health Problems of Aged People. Int J Res Soc Sci.

[5] Help age internationale (2011). A study of older peoples livelihoods in.

[6] EDHS: CSA (2014). Population stablization report Ethiopia.

[7] Help age Internationle (2013). The State of Health and Ageing in Ethiopia : A Survey of Health Needs and Challenges of Service Provisions Help Age International helps older people be empowered.

[8] Lemma S (2014). Care and support to the elderly.

[9] Dikken J (2017). How to measure nurses’ knowledge and attitude regarding older patients.

[10] Elebiary H, Hend Elshenewy SA (2018). Knowledge and attitudes of nurses towards caring of elderly people in health care sitting. J Nurs Heal Sci.

[11] WHO (2019). World Population Ageing.

[12] Muhsin AA, Munyogwa MJ, Kibusi SM (2020). Poor level of knowledge onelderly care despite positive attitude among nursing students in Zanzibar Island: findings from across-sectionalstudy. BMC Nurs.

[13] Liu YE, Norman IJ, While AE (2013). Nurses’ attitudes towards older people: a systematic review. Int J Nurs Stud.

[14] Faronbi JO, Adebowale O, Faronbi GO, O.O. Musa JA. (2017). Perception, Knowledge and Attitude of Nursing Students towards the Care of Older Patients. Int J Afr Nurs Sci.

[15] Eltantawy SHAE (2013). Relation between Nursing Students’ Knowledge of Aging and Attitude towards Elderly People and Their Will and Intent to Work with the Elderly. J Educ Pract.

[16] Argaw Z, Tamiru S, Ayalew Y, Habte T. Knowledge and Attitude towards Care of Elder Patients and Associated Factors among Nurses Working in Government Hospitals in Addis Ababa, Ethiopia. Int J Sci Eng Res. 2019;9(12):1-14.

[17] Paulo J, da Silva AL, Sá-Couto P, Marie Boltz EC (2015). Portuguese nurses ’ knowledge of and attitudes toward hospitalized older adults. Scand J Caring Sci.

[18] Mansouri Arani M, Aazami S, Azami M, Borji M. Assessing attitudes toward elderly among nurses working in the city of Ilam. Int J Nurs Sci. 2017;4(3):311-3. 10.1016/j.ijnss.2017.06.009. 10.1016/j.ijnss.2017.06.009 PMC6626171 31406758

[19] World Health Organization (2017). Global strategy and action plan on ageing and health.

[20] Mattos MK, Jiang Y, Seaman JB, Nilsen ML, Chasens ER, Novosel LM (2015). Bacc alaureate nursing students’ knowledge of and attitudes toward older adults. J Gerontol Nurs.

[21] Salmond SW, Echevarria M (2017). Healthcare transformation and changing roles for nursing. Orthop Nurs.

[22] Dikken J, Hoogerduijn JG, Kruitwagen C, Schuurmans MJ (2016). Content validity and psychometric characteristics of the “knowledge about older patients quiz” for nurses using item response theory. J Am Geriatr Soc.

[23] Dikken J, Hoogerduijn JG, Klaassen S, Lagerwey MD, Shortridge-Baggett L, Schuurmans MJ (2017). The knowledge-about-older-patients-quiz (KOP-Q) for nurses: cross-cultural validation between the Netherlands and United States of America. Nurse Educ Today.

[24] Dikken J, Hoogerduijn JG, Schuurmans MJ (2015). Construct development, description and initial validation of the knowledge about older patients quiz (KOP-Q) for nurses. Nurse Educ Today.

[25] Oyetunde MO, Ojo LYO OO (2013). Nurses ’ attitude towards the care of the elderly : implications for gerontological nursing training. J Nurs Educ Pract.

[26] Grady PA. Advancing the health of our aging population: a lead role for nursing science. Nursing outlook. 2011;59(4):207–9. 10.1016/j.outlook.2011.05.017. 10.1016/j.outlook.2011.05.017 PMC3197709 21757076

[27] Zhang H (2019). Knowledge , attitude and self- efficacy of elderly caregivers in Chinese nursing.

[28] Dikken J, Bakker A, Hoogerduijn JG, Schuurmans MJ (2018). Comparisons of knowledge of Dutch nursing students and hospital nurses on aging. J Contin Educ Nurs.

[29] Engström G, Ingegerd Fagerberg E (2018). Attitudes towards older people among Swedish health care students and health care professionals working in elder care. Nurs Rep 2018.

